# Long-chain n-3 and n-6 polyunsaturated fatty acids and risk of atrial fibrillation: Results from a Danish cohort study

**DOI:** 10.1371/journal.pone.0190262

**Published:** 2017-12-22

**Authors:** Lotte Maxild Mortensen, Søren Lundbye-Christensen, Erik Berg Schmidt, Philip C. Calder, Mikkel Heide Schierup, Anne Tjønneland, Erik T. Parner, Kim Overvad

**Affiliations:** 1 Department of Public Health, Section for Epidemiology, Aarhus University, Aarhus, Denmark; 2 Department of Cardiology, Aalborg AF Study Group, Aalborg University Hospital, Aalborg, Denmark; 3 Human Development and Health Academic Unit, Faculty of Medicine, University of Southampton, Southampton, United Kingdom; 4 National Institute for Health Research Southampton Biomedical Research Centre, University Hospital Southampton NHS Foundation Trust and University of Southampton, Southampton, United Kingdom; 5 Bioinformatics Research Center, University of Aarhus, Aarhus C, Denmark; 6 Danish Cancer Society Research Center, Unit of Diet, Genes and Environment, Copenhagen, Denmark; 7 Department of Public Health, Section for Biostatistics, Aarhus University, Aarhus, Denmark; The Pennsylvania State University, UNITED STATES

## Abstract

**Background:**

Studies of the relation between polyunsaturated fatty acids and risk of atrial fibrillation have been inconclusive. The risk of atrial fibrillation may depend on the interaction between n-3 and n-6 polyunsaturated fatty acids as both types of fatty acids are involved in the regulation of systemic inflammation.

**Objective:**

We investigated the association between dietary intake of long chain polyunsaturated fatty acids (individually and in combination) and the risk of atrial fibrillation with focus on potential interaction between the two types of polyunsaturated fatty acids.

**Design:**

The risk of atrial fibrillation in the Diet, Cancer and Health Cohort was analyzed using the pseudo-observation method to explore cumulative risks on an additive scale providing risk differences. Dietary intake of long chain polyunsaturated fatty acids was assessed by food frequency questionnaires. The main analyses were adjusted for the dietary intake of n-3 α-linolenic acid and n-6 linoleic acid to account for endogenous synthesis of long chain polyunsaturated fatty acids. Interaction was assessed as deviation from additivity of absolute association measures (risk differences).

**Results:**

Cumulative risks in 15-year age periods were estimated in three strata of the cohort (N = 54,737). No associations between intake of n-3 or n-6 long chain polyunsaturated fatty acids and atrial fibrillation were found, neither when analyzed separately as primary exposures nor when interaction between n-3 and n-6 long chain polyunsaturated fatty acids was explored.

**Conclusion:**

This study suggests no association between intake of long chain polyunsaturated fatty acids and risk of atrial fibrillation.

## Introduction

Atrial fibrillation (AF) is characterized by an irregular electrical activity of the atria leading to disturbed coordination of electric impulses from the atria to the ventricles and thereby reducing the ventricular pump function.

AF is the most common cardiac arrhythmia with a prevalence that increases with age to ultimately affect at least 8% of those older than 80 years [1]. AF patients have a substantially elevated risk of stroke and premature death [2] and often a reduced quality of life due to cardiac symptoms like palpitations, dyspnea and reduced working capacity [3]. AF is a multi-factorial disease with several contributing risk factors including other cardiac conditions, heredity, age, obesity, extreme physical activity, alcohol intake, and various comorbidities [4]. Systemic inflammation has also been suggested to be associated with AF [4–6]. Thus, studies have shown that the serum levels of C-reactive protein, tumor necrosis factor-α and interleukin 6, all markers of systemic inflammation, are higher among patients with AF compared with controls [6, 7]. These findings were supported by a meta-analysis [8] which concluded that C-reactive protein and interleukin 6 were positively associated with risk of AF in the general population.

Systemic inflammation is partly regulated by a group of lipid signaling molecules, the eicosanoids and docosanoids, which are synthesized from long chain polyunsaturated fatty acids (LC-PUFAs). Thus, these mediators of inflammation may be a possible link between PUFAs and the risk of AF. PUFAs comprise two main families, the n-3 PUFAs and the n-6 PUFAs, with subtypes of varying carbon chain length (mainly C18-C22) and degree of unsaturation. The n-3 LC-PUFAs (C20-C22) are obtained directly from the diet (primarily through seafood) and by endogenous synthesis from the plant derived n-3 PUFA α-linolenic acid (α-LA) which to a limited extent can be metabolized to LC-PUFAs, mainly eicosapentaenoic acid (EPA) [9]. In a similar way, the n-6 LC-PUFA arachidonic acid (AA) is synthesized by conversion of linoleic acid (LA) [9, 10]. After incorporation into cells and tissues, the LC-PUFAs can be further metabolized into eicosanoids and docosanoids in metabolic pathways catalyzed by shared enzymes. Bioavailability of n-3 LC-PUFAs affects the amount of n-6 derived mediators, and competition between the two LC-PUFA families regarding the synthesis of the mediators has been observed [11]. In general, the n-6 derived mediators are more pro-inflammatory than the n-3 derived ones [12]. This suggests that possible inflammatory effects due to the intake of n-3 PUFAs may be affected by the intake of n-6 PUFAs and vice versa.

Epidemiologic and clinical studies of the association between n-3 LC-PUFAs and risk of AF have shown divergent results [13, 14]. When focusing on cohort studies of fish or n-3 LC-PUFAs obtained from the diet and AF occurring without prior cardiovascular surgery, findings have also been mixed, although the majority of studies have shown no association [15–21]. To our knowledge, no studies of n-6 PUFAs and AF risk have been published. Further, in a recent Cochrane review of the role of n-6 PUFAs in the primary prevention of cardiovascular disease including AF [22], no studies with AF as outcome were included.

The overall aim of the present work was to study the association between LC-PUFAs and risk of AF. With respect to incident AF, we explored the individual associations for the n-3 and n-6 LC PUFAs as well as their potential interaction.

## Methods

### Study population

The data source for this study was the Danish Diet, Cancer and Health cohort which has been described in detail elsewhere [23]. This cohort contains data from 57,053 participants born in Denmark and living in the urban areas of Copenhagen and Aarhus at enrolment. Participants were enrolled from December 1993 to May 1997 when they were between 50 and 65 years old. The participants gave informed written consent including permission for prospective data collection from national registries. In the present study, participants with AF, atrial flutter (AFL), myocardial infarction, heart failure or cancer before recruitment were excluded. The participants gave informed written consent including permission for prospective data collection from national registries. The Diet, Cancer, and Health cohort study has been approved by the Health Research Ethics, the Capital Region of Denmark and the Danish Data Protection Agency.

### Baseline information, exposure and outcome assessment

At baseline, the participants filled in a detailed, previously validated, semi-quantitative food frequency questionnaire (FFQ) with 192 items including 24 questions regarding intake of fish and food products containing fish. This information was used to calculate the intake of fatty acids by use of Danish food composition tables and the software FoodCalc [24] For this study, intake information for the following n-3 PUFAs was calculated: α-LA (18:3), EPA (20:5), DPA (22:5) and DHA (22:6). For n-6 PUFAs, LA (18:2) and AA (20:4) were calculated. Additionally, the participants answered questions about health, lifestyle, and medications. In order to minimize errors, an interviewer reviewed the questionnaires together with the participant at the baseline visit [23]. The outcome, denoted AF throughout this article, was incident AF and/or AFL during follow-up without preceding myocardial infarction or heart failure. Relevant diagnoses were extracted from the Danish National Patient Registry by cross-linking civil registration numbers. The diagnoses were recorded using the Eighth International Classification of Diseases (ICD-8) until the end of 1993 (AF (427.93) and AFL (427.94) in the Danish version which is equivalent to AF or AFL (427.4) in the international version). From January 1994, the ICD-10 classification was used with the diagnosis of AF and/or AFL (I.48).

### Statistical analysis

Data were analyzed as time-to-event data using the pseudo-observation method [25]. In order to estimate cumulative risks in age periods, age was chosen as the underlying time scale. The risk of AF was analyzed in three separate strata based on baseline age tertiles. The three strata resulted in three15-year age periods for cumulative risk: age 50–65, age 55–70 and age 60–75 years. The decision of estimating cumulative risk in different (partly overlapping) age periods was based on biological arguments. Age is a strong risk factor for AF[1], pointing at changes of the underlying biology with age. So, in order to minimize the presence of different biological mechanisms in the same analyses, separate risk estimates for different age groups seemed reasonable. The specific choice of 15-year age frames was determined by the follow-up time in the cohort. Participants entered the cohort at baseline and were followed until emigration, myocardial infarction, heart failure, death, AF diagnosis, administrative end of follow-up on December 30, 2009, or to the age of the upper age frame (age 65, 70, and 75 years respectively for stratum 1, 2, and 3). We prioritized to end the observation time by the end of 2009 to limit the time distance between sampling of exposure information and end of follow-up. At that point in time, the selected analysis age range (age 50–75 years) was covered adequately. Death, myocardial infarction and heart failure during follow-up were treated as competing risks. According to the complete-case approach and under the assumption of ‘missing completely at random’, we excluded participants for whom data regarding one or more covariates were missing. The analyses were adjusted for baseline information about the following potential confounders selected *a priori*: sex, baseline age (as a proxy for time since sampling of exposure variables), intake of fish oil capsules, smoking, alcohol intake, BMI, waist circumference, angina pectoris, diabetes, intake of α-LA and LA. Continuous covariates were modeled as restricted cubic splines with 3 knots (placed at the 10, 50 and 90 percentiles) [26]. Based on *a priori* knowledge, we did not include any of the confounders as interaction terms in the model. The intake of n-3 LC-PUFAs and n-6 LC-PUFAs was expressed in tertiles resulting in 9 exposure groups in the interaction analysis. Association analyses were carried out with n-3 LC-PUFAs as primary exposure, n-6 LC-PUFAs as primary exposure, and the interaction between n-3 and n-6 LC-PUFAs as main analyses. Low intake tertile groups were used as reference. We analyzed data on an additive risk scale; hence, associations are expressed as risk differences (RDs). Interaction was assessed as deviation from additivity of absolute measures of association (risk differences).

The assumptions of strongly independent entry and censoring were checked, as were the assumption of independency between distribution of entry time and covariates and the assumption of independency between time of censoring and covariates. Data were analyzed using Stata Statistical Software (Stata 13) [27].

## Results

**Fig 1:**
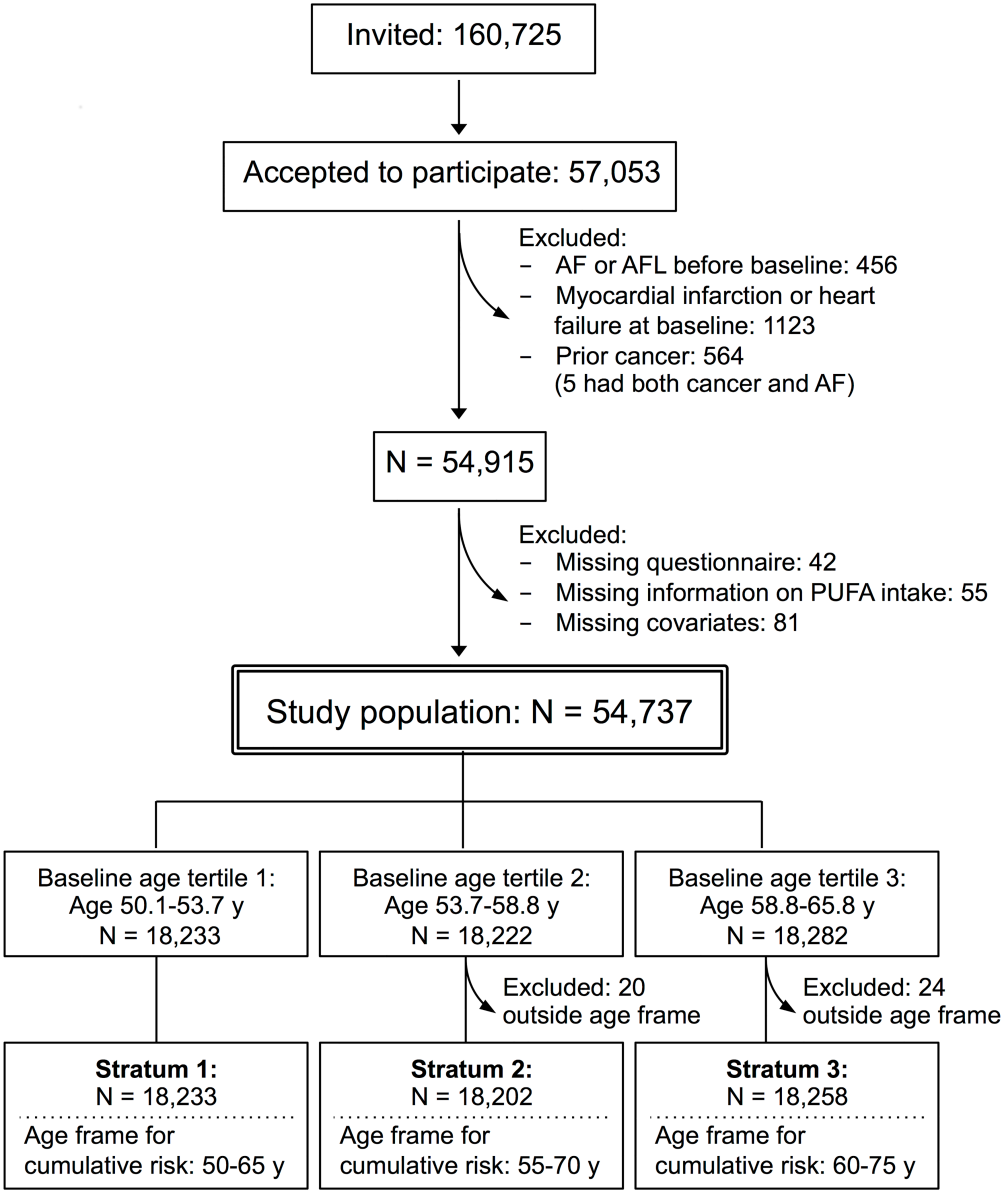
Flowchart of the basic study population from the Diet, Cancer and Health cohort. AF: atrial fibrillation, AFL: Atrial flutter, PUFA: polyunsaturated fatty acid.

The final study population comprised 54,737 men and women aged 50 to 65 years at enrolment. Due to cohort heterogeneity and violations of the assumptions behind the pseudo-observation method, all analyses were carried out in three separate strata defined by baseline tertiles of age and, consequently, three age frames for cumulative risks. Hence, strata-defined study populations, strata 1, 2, and 3, consisted of 18,233, 18,202, and 18,258 participants (in strata 2 and 3, 20 and 24 participants left the study before commencement of the observation time at age 55 and 60 years, respectively) (Fig 1).

During follow-up, 2,274 participants within the selected age frames were diagnosed with AF. A total of 337 participants were lost to follow-up due to emigration or change of personal identification number, and 7,276 participants left the study due to competing risks (myocardial infarction, heart failure or death). At the administrative end of follow-up on 30 December 2009, 18,598 participants were still at risk within the analysis age frames, while 26,208 participants reached the upper age frames specific for each stratum without any events (AF or competing risks). The median follow-up time was 13.5 years. A summary of the distribution of events for each stratum can be found in S1 Table. Baseline characteristics for each stratum are presented in Table 1.

**Table 1:** Baseline characteristics of the study population from the Diet, Cancer and Health cohort.

Characteristics	STRATUM 1 (N = 18,233)		STRATUM 2 (N = 18,202)		STRATUM 3 (N = 18,258)	
	Cohort	Cases	Cohort	Cases	Cohort	Cases
N	18233	487	18202	705	18258	1082
Baseline age (y)	51.8 (50.6, 53.3)^a^	51.8 (50.6, 53.2)	56.1 (54.1, 58.2)	56.1 (54.1, 58.2)	61.8 (59.3, 64.5)	61.8 (59.3, 64.4)
Sex						
Men (%)	48.2	69.2	47.2	66.8	45.2	56.2
Women (%)	51.8	30.8	52.8	33.2	54.8	43.8
BMI (kg/m^2^)	25.3 (21.2, 31.0)	26.4 (21.8, 33.4)	25.5 (21.5, 31.0)	26.0 (22.0, 33.3)	25.8 (21.6, 31.3)	26.8 (22.2, 32.8)
Waist (cm)	88 (72, 104)	94 (75, 112)	88 (72, 105)	93 (76, 110)	89 (73, 105)	94 (76, 110)
Alcohol (g/day)	13.7 (1.8, 50.4)	17.9 (2.3, 65.3)	12.9 (1.6, 47.2)	16.2 (2.1, 60.8)	12.2 (1.4, 45.4)	15.3 (1.8, 56.3)
Smoking						
Never (%)	37	36	37	32	33	33
Former (%)	26	24	27	31	32	33
Current < 15 CPD (%)	12	12	13	12	14	12
Current 15–25 CPD (%)	17	18	16	16	16	17
Current > 25 CPD (%)	8	10	7	9	5	5
Fish oil supplement (%)	13.2	13.1	17.1	18.3	19.9	18.5
Angina pectoris, self reported (%)	1.2	3.7	2.1	4.0	3.1	4.1
Diabetes, self reported (%)	1.5	2.3	1.9	1.8	2.7	3.8
Hypertension, self reported (%)	12.6	18.3	15.4	20.0	19.5	27.9
Intake of α-LA (g/day)	1.7 (1.0, 2.9)	1.8 (1.0, 3.2)	1.7 (1.0, 2.9)	1.8 (1.1, 3.0)	1.7 (1.0, 2.9)	1.7 (1.0, 2.9)
Intake of LA (g/day)	10.9 (6.2, 18.0)	11.4 (6.0, 19.1)	10.5 (6.0, 17.7)	10.9 (6.4, 18.9)	10.6 (6.1, 17.9)	10.5 (6.0, 18.1)

CPD: cigarettes per day, α-LA: α-linolenic acid, LA: linoleic acid

^a^Median; 80% central range in parentheses (all such values)

In all strata, cases had a higher median BMI and waist circumference and a higher intake of alcohol compared with the total cohort. In general, a higher prevalence of comorbidity was seen among cases. The distribution of LC-PUFA intake for each stratum is presented in Table 2. For n-3 LC-PUFAs, the median intake varied between strata. There were only minor differences in the distribution of intake of n-6 LC-PUFAs between the different strata.

**Table 2:** Distribution of the dietary intake of n-3 and n-6 LC-PUFAs.

	**STRATA**^a^		
	Stratum 1	Stratum 2	Stratum 3
	(N = 18,233)	(N = 18,202)	(N = 18,258)
n-3 LC-PUFAs	0.59 (0.26, 1.18)	0.62 (0.28, 1.25)	0.67 (0.29, 1.34)
n-6 LC-PUFAs	0.10 (0.05, 0.18)	0.10 (0.05, 0.18)	0.10 (0.05, 0.17)

LC-PUFAs are given as median intake (g/d) with 80% central range in parentheses

^a^Stratum 1, 2 and 3 were defined by baseline age tertiles

According to the CIs, no consistent nor statistically significant associations were found when n-3 LC-PUFAs and n-6 LC-PUFAs were modeled as the primary exposure (Tables 3 and 4).

**Table 3:** Intake of n-3 LC-PUFAs and risk of AF.

	N-3 LC-PUFA, tertiles^a^	STRATA^b^		
		Stratum 1	Stratum 2	Stratum 3
		(age 50–65 y)	(age 55–70 y)	(age 60–75 y)
MODEL 1^c^	1	REF	REF	REF
	2	0.07 (-0.5, 0.7)	-0.26 (-1.0, 0.5)	-0.90 (-1.8, 0.1)
	3	0.45 (-0.2, 1.1)	0.33 (-0.5, 1.2)	-0.25 (-1.2, 0.8)
MODEL 2^d^	1	REF	REF	REF
	2	0.18 (-0.4, 0.8)	-0.17 (-1.0, 0.6)	-0.66 (-1.6, 0.3)
	3	0.67 (-0.04, 1.4)	0.53 (-0.4, 1.4)	0.26 (-0.8, 1.3)

Risk of AF is given by age-specific cumulative risk differences (RD in %) with 95% CI in parentheses

^a^Tertiles of intake of n-3 LC-PUFAs (EPA, DPA and DHA)
Category boundaries for the tertiles, T1-T3 (g/d): Stratum 1, T1(0–0.46) T2(0.46–0.75) T3(0.75–5.28) Stratum 2, T1(0–0.49) T2(0.49–0.79) T3(0.79–6.35) Stratum 3, T1(0–0.52) T2(0.52–0.86) T3(0.86–7.22)

^b^Stratum 1, 2 and 3 were defined by baseline age tertiles

^c^Model 1 included baseline age, sex, BMI, waist circumference, alcohol intake, smoking, fish oil supplements, angina pectoris, diabetes, and hypertension

^d^Model 2 included variables in model 1 and intake of α-LA

**Table 4:** Intake of n-6 LC-PUFAs and risk of AF.

	N-6 LC-PUFA, tertiles^a^	STRATA^b^		
		Stratum 1	Stratum 2	Stratum 3
		(age 50–65 y)	(age 55–70 y)	(age 60–75 y)
MODEL 1^c^	1	REF	REF	REF
	2	-0.34 (-0.9, 0.3)	-0.21 (-1.0, 0.6)	-0.66 (-1.6, 0.3)
	3	0.18 (-0.5, 0.9)	0.01 (-0.9, 0.9)	-0.51 (-1.5, 0.5)
MODEL 2^d^	1	REF	REF	REF
	2	-0.26 (-0.9, 0.3)	-0.22 (-1.0, 0.6)	-0.41 (-1.3, 0.5)
	3	0.27 (-0.4, 1.0)	-0.07 (-1.0, 0.9)	-0.10 (-1.2, 1.0)

Risk of AF is given by age-specific cumulative risk differences (RD in %) with 95% CI in parentheses

^a^Tertiles of intake of n-6 LC-PUFAs (AA)
Category boundaries for the tertiles, T1-T3 (g/d): Stratum 1, T1(0–0.08) T2(0.08–0.12) T3(0.12–0.75) Stratum 2, T1(0–0.08) T2(0.08–0.12) T3(0.12–0.81) Stratum 3, Stratum 3, T1(0–0.08) T2(0.08–0.12) T3(0.12–0.90)

^b^Stratum 1, 2 and 3 were defined by baseline age tertiles

^c^Model 1 included baseline age, sex, BMI, waist circumference, alcohol intake, smoking, fish oil supplements, angina pectoris, diabetes, and hypertension

^d^Model 2 included variables in model 1 and intake of LA

In Table 5, the RDs for the combined n-3/n-6 PUFA tertile analyses are shown with the low intake n-3 and n-6 LC-PUFA tertiles as reference. It should be noted that the analyses were performed for each stratum providing 3 parallel results which are all presented in the table. Boldface states the magnitude of the interaction assessed as deviation from additivity of the risk differences in the individual exposure groups. Thus, in the analysis of stratum 1, the observed RD in the joint n-3 and n-6 LC-PUFA high intake tertile group (the participants that belong to third intake tertile regarding both PUFAs) was 0.70 (−0.4, 1.8)% and the interaction was assessed to 0.89 (−1.0, 2.7)% calculated as deviation of the observed 0.70% from the sum of the RDs in the individual exposure groups (−0.19%, calculated as the sum of −0.56% and 0.37%). Consistent for all three strata, no substantial interaction between n-3 and n-6 LC-PUFAs was found (Table 5). The point estimates (RDs in percent) were close to zero, and all confidence intervals included zero with no consistent direction of the point estimates.

**Table 5:** N-3 and n-6 LC-PUFAs and risk of AF.

		STRATA^a^								
		Stratum 1 (age 50–65 y)			Stratum 2 (age 55–70 y)			Stratum 3 (age 60–75 y)		
		n-6 LC-PUFA tertiles			n-6 LC-PUFA tertiles			n-6 LC-PUFA tertiles		
		1	2	3	1	2	3	1	2	3
n-3 LC-PUFA tertiles	1	REF	-0.57 (-1.5, 0.4)	-0.56 (-1.8, 0.7)	REF	-0.07 (-1.3, 1.2)	0.54 (-1.2, 2.3)	REF	-0.63 (-2.1, 0.8)	2.07 (-0.3, 4.5)
	2	-0.27 (-1.1, 0.6)	-0.11 (-1.0, 0.8)	0.20 (-0.9, 1.3)	-0.14 (-1.3, 1.0)	-0.32 (-1.5, 0.8)	0.04 (-1.3, 1.4)	0.11 (-1.4, 1.6)	**-**0.89 (-2.3, 0.5)	-0.54 (-2.1, 1.0)
			**0.73 (-0.6, 2.1)**	**1.03 (-0.6, 2.7)**		**-0.11 (-1.9, 1.6)**	**-0.36 (-2.6, 1.9)**		**-0.37 (-2.5, 1.8)**	**-2.72 (-5.6, 0.2)**
	3	0.37 (-0.9, 1.6)	**-**0.07 (-1.1, 0.9)	0.70 (-0.4, 1.8)	1.27 (-0.5, 3.0)	0.48 (-0.8, 1.8)	0.23 (-1.1, 1.5)	0.64 (-1.3, 2.6)	1.03 (-0.6, 2.7)	-0.03 (-1.5, 1.5)
			**0.13 (-1.5, 1.8)**	**0.89 (-1.0, 2.7)**		**-0.72 (-2.9, 1.5)**	**-1.58 (-4.1, 0.9)**		**1.03 (-1.5, 3.6)**	**-2.74 (-5.8, 0.3)**

Risk of AF is given by age-specific cumulative risk differences (RD in %) with 95% CI in parentheses. With boldface is given estimates of interaction assessed as deviation from additivity of the RDs as explained in detail in the main text. The estimates were adjusted for baseline age, sex, BMI, waist circumference, alcohol intake, smoking, fish oil supplements, angina pectoris, diabetes, hypertension, and intake of α-LA and LA

^a^The strata were defined by baseline age tertiles. For each stratum cumulative risk is indicated for is measured in strata-specific 15 years age frames (given in parentheses)

The absolute risks were estimated for male and female reference individuals from the first LC-PUFA intake tertiles (Table 6), showing that, generally, the risk increased with age. The higher risk according to age appeared later for women than for men. Also, male reference individuals appeared to be at higher risk compared with the female reference individuals.

**Table 6:** Absolute risks for a female and male reference individuals.

Sex	STRATA^a^		
	Stratum 1	Stratum 2	Stratum 3
	(age 50–65 y)	(age 55–70 y)	(age 60–75 y)
Female	1.02 (0.2, 1.9)	1.31 (0.2, 2.4)	4.24 (2.9, 5.6)
Male	3.74 (2.7, 4.8)	5.14 (3.8, 6.5)	7.64 (6.0, 9.2)

Absolute cumulative risks in strata-specific 15 years age frames with 95% CIs in parentheses. Characteristics for reference individuals were: Median age (age 51.8, 56, 61.8 years in stratum 1, 2, and 3), median intake of α-LA and LA, lowest PUFA intake tertiles, no smoking, no intake of fish oil capsules, no comorbidity, BMI at 25. Sex specific reference values: Waist circumference at 80 cm (women) and 94 cm (men). An alcohol intake at 12 g/day (women) and 24 g/d (men). For BMI, waist circumference and alcohol intake the reference values are given by the maximum limit recommended by The Danish Health Authority

^a^The strata were defined by baseline age tertiles. For each stratum cumulative risk is indicated for strata-specific 15 years age frames (given in parentheses)

## Discussion

This study indicated no association between intake of n-3 and n-6 LC-PUFAs and risk of incident AF. The findings were consistent for all strata for both n-3 and n-6 LC-PUFAs measured as primary exposures and for the analyses of potential interaction between them.

Selection at recruitment (35% participation, Fig 1) is unlikely to have affected the findings of this association analysis as we expect the mechanisms of action of the dietary LC-PUFAs to be unaffected by potential selection of the participants at enrolment. Selection bias during follow-up is also unlikely to have affected the analyses since it was explicitly tested, as a part of the model control, if participants administratively censored during follow-up were different in terms of event risk or covariate distribution compared with the corresponding participants remaining in the study. The analysis was designed in order to avoid this, thereby minimizing this source to potential selection problems during follow-up. Random measurement error could not be fully avoided as the exposure and covariate assessments relied on self-reported information from FFQs. Modeling the exposure as a categorical variable (i.e. as tertiles) could have led to information problems in terms of categorizing the participants to wrong exposure groups. As it seemed unlikely that these sources of potential misclassification would be related to the future diagnosis of AF, a true association could have been concealed due to a bias towards the null hypothesis. Information problems in connection with an AF diagnosis are not likely as this information was obtained by linkage of civil registration numbers with the Danish National Patient Registry. The diagnoses have previously been validated with a positive predictive value above 92% [28]. The presence of residual confounding, either as a consequence of the self-reported information on covariates or because of risk factors not taken into account, cannot be ruled out. We did not include data on physical activity in these analyses. AF has only been associated with *extreme* physical activity [4] and as it was uncommon in Denmark in the 1990ties that people aged 50–65 years practiced extreme sports, we assess this potential confounder to be of minor concern. Overall, the impact of the adjustment for potential confounding was weak.

Our findings of no association between intake of n-3 LC-PUFAs and AF risk are consistent with the majority of the previous studies of dietary intake of n-3 LC-PUFAs [16–19, 21]. These studies do not support the findings of Mozaffarian *et al*. [15] based on a cohort study with 980 incident cases among 4815 men and women (> 65 years of age) followed for 12 years. They reported an inverse association between the intake of fish (baked or boiled) and risk of incident AF, but did not find any associations between the intake of fried fish and AF. However, our exposure assessment was not identical as we recalculated the FFQ information to PUFA intake (g/d) in contrast to weekly number of servings and also, we did not stratify on cooking method. Our findings also contradict the findings of a cohort study (3284 incident AF cases) by Rix *et al*. [20] reporting a U-shaped association between intake of n-3 LC-PUFA and AF with the lowest risk near the median intake of n-3 LC-PUFA (630 mg/day). This study was based on the same cohort data as ours but different analysis designs interfere with the comparability. A major difference was that our outcome definition was restricted to AF without prior diagnoses of myocardial infarction and heart failure. To our knowledge, no studies have investigated n-3 LC-PUFAs and AF taking into account potential biological interaction with n-6 LC-PUFAs.

We designed this study with focus on the metabolism of the PUFAs. One aspect was the analyses of potential interaction between n-3 and n-6 LC-PUFAs. Another aspect was to take the potential endogenous contribution of LC-PUFAs into account. This was addressed by adjusting for intake of α-LA and LA as an attempt to rule out a potential effect from this source of LC-PUFAs. This may have been a too simplified solution, as the individual conversion of α-LA and LA to their LC-PUFA derivatives is affected by factors other than the intake of substrate, e.g. intake of other fatty acids and genetics, implying individual pathway efficiency [29–34]. Although the general consensus is that the conversion of C18 PUFAs to the LC-PUFAs is poor [35, 36], which is often the argument for not taking α-LA and LA into further consideration, it may still be relevant to look further into this in future studies. This was also suggested by Madden *et al*. with regard to the genetic polymorphisms involved in the PUFA conversion pathway [37]. The n-6 PUFA substrate, LA, is the most abundant dietary PUFA, and the dietary intake of AA is very limited. Consequently, the dietary substrate-product ratio (LA:AA) is high. Thus, even for limited conversion percentages, the endogenous contribution could be relatively large compared with the direct dietary intake of n-6 LC-PUFA. Considering n-3 PUFAs, the substrate-product ratio (α-LA:EPA) is not as large as in the n-6 PUFA family. However, in cases of low n-3 LC-PUFA intake and high α-LA intake (e.g. a vegetarian diet rich in walnuts, canola oil or flaxseed oil), the endogenous contribution can be substantial. In future FFQ-based studies, it could be relevant to identify the factors that affect the conversion (genetic variation, product- and feedback inhibition, competing substrate), and either estimate the endogenous contribution or model the factors as interacting terms in the analyses.

There is an ongoing discussion about whether the underlying statistical model should be additive or if relative measures from a multiplicative model are useful for evaluation of biological interaction, e.g. by use of Relative Excess Risk due to Interaction (RERI) as summary analysis [38–42]. Here we analyzed the biologically substantiated interaction between n-3 and n-6 LC-PUFAs on an additive risk scale as deviation from additivity of risk differences [43]. For that purpose, we used a relatively new statistical tool, the pseudo-observation method [44, 45] which is an alternative to, for example, Cox regression in terms of analyzing time-to-event data. In contrast to Cox regression, the pseudo-observation method makes it possible to choose between relative and absolute measures of association, hence it enables an assessment of potential interaction on an additive risk scale which is most important from a public health point of view.

In conclusion, despite the limitations mentioned above, this study showed a consistent lack of association in all strata, pointing at no clinically relevant influence of intake of LC-PUFAs on the development of AF in a target Western population represented by the Danish Diet, Cancer and Health study population.

## Supporting information

S1 Table Summary of follow-up outcomes and follow-up time shown for each strata. (DOCX)
