# Supplementary material for: Long-chain n-3 and n-6 polyunsaturated fatty acids and risk of atrial fibrillation: Results from a Danish cohort study
Source: PLoS One. 2017 Dec 22;12(12):e0190262. doi: 10.1371/journal.pone.0190262 (PMC5741257; doi:10.1371/journal.pone.0190262)
Supplement: S1 Table — (DOCX) [file pone.0190262.s001.docx]

**Supporting information**

**S1 Table. Summary of follow-up outcomes and follow-up time shown for each strata**

|  |  |  |  | **STRATA^a^** |  |  |
| --- | --- | --- | --- | --- | --- | --- |
|  |  | Stratum 1 (N=18,233) |  | Stratum 2 (N=18,202) |  | Stratum 3 (N=18,258) |
| AF (N) |  | 487 |  | 705 |  | 1,082 |
| Competing risk event^b^ (N) |  | 1,563 |  | 2,284 |  | 3,429 |
| Administrative censoring (N) |  | 5,268 |  | 7,885 |  | 5,445 |
| Loss-to-follow up censoring (N) |  | 134 |  | 136 |  | 67 |
| Free of events^c^ by the age of the upper age frame^d^ (N) |  | 10,781 |  | 7,192 |  | 8,235 |
| Median follow-up time (y) |  | 13.5 |  | 13.5 |  | 13.3 |

^a^Stratum 1, 2 and 3 were defined by baseline age tertiles

^b^Death, myocardial infarction, heart failure

^c^AF, death, myocardial infarction, heart failure

^d^Age 65, 70 and 75 y, respectively for strata 1, 2 and 3
